# Context of use matters: interpreting extracellular vesicle TDP-43 as a biomarker in ALS

**DOI:** 10.1186/s40478-026-02376-x

**Published:** 2026-07-11

**Authors:** Jenna M. Gregory

**Affiliations:** Institute of Medical Sciences, University of Aberdeen, Aberdeen, AB25 2ZD UK

**Keywords:** Amyotrophic lateral sclerosis, TDP-43, Neuron-derived extracellular vesicles, Biomarkers, Surrogate endpoints, Pharmacodynamic biomarkers, Clinical trials, Neuropathology

The recently reported Phase 2 PRIME-C data in ALS [1] have generated understandable excitement, particularly around the observation that neuron-derived extracellular vesicle (NDE) TDP-43 levels were reduced following treatment. Any biological signal in ALS is worth attention. However, as pathologists, we should be careful not to confuse an interesting biomarker observation with evidence of disease modification.

The key question is simple: is lower NDE-derived TDP-43 a good thing? At present, we do not know.

TDP-43 pathology is central to ALS. Mislocalisation, aggregation, and loss of normal nuclear function are among the defining pathological features of the disease. However, the relationship between intracellular TDP-43 pathology and extracellular TDP-43 measured in neuron-derived vesicles remains poorly understood. Critically, there is no established evidence that changes in extracellular vesicle-associated TDP-43 reflect treatment response, disease progression, or improvement in underlying pathology. In fact, there are multiple biologically plausible explanations for the observed reduction. One possibility is that treatment reduces pathological release of TDP-43-containing vesicles, reflecting a beneficial effect. But an equally plausible explanation is that TDP-43 is becoming more insoluble and increasingly retained within cells, leading to lower extracellular levels while intracellular pathology worsens. Similarly, if extracellular TDP-43 were to increase, that could reflect greater neuronal injury, or alternatively enhanced clearance of pathological protein species. The point is that the direction of change alone tells us very little. At this stage, neither an increase nor a decrease can be confidently interpreted as favourable or unfavourable. This highlights a common challenge in biomarker development: detection biomarkers are not necessarily treatment-response biomarkers.

Inflammatory bowel disease provides a useful example. Faecal calprotectin is widely used to monitor disease activity and treatment response because years of research have demonstrated a clear relationship between calprotectin levels and intestinal inflammation. Its context of use has been established through rigorous clinical validation. TDP-43 in neuron-derived extracellular vesicles has not yet undergone that process. It remains an exploratory biomarker. That does not diminish its value. Exploratory biomarkers are essential for understanding disease biology and drug mechanisms. However, their role is to generate hypotheses, not confirm efficacy. Context of use matters enormously in biomarker science. A biomarker may be useful for detecting disease but not for monitoring response. It may indicate target engagement without demonstrating clinical benefit. It may correlate with pathology without being directly related to disease progression. At present, NDE-derived TDP-43 sits firmly within the exploratory category. It is entirely appropriate to measure it and investigate whether changes correlate with clinical outcomes, imaging findings, or future pathological data. What is not yet justified is treating changes in this biomarker as evidence that disease pathology has improved.

There is another important consideration. The intervention studied in PRIME-C is not restricted to the central nervous system, it is multi-organ penetrant, which complicates interpretation of peripheral biomarker changes. Neurons are not exclusive to the brain and spinal cord. The peripheral and autonomic nervous systems extend throughout the body, innervating virtually every organ system. As a result, circulating neuron-derived extracellular vesicles may reflect biological processes occurring across multiple organs. As we know that TDP-43 pathology can affect other organs, rescuing this may be therapeutically beneficial but how this relates to disease progression of ALS and how a treatment that affects multiple tissues could alter extracellular vesicle biology, protein trafficking, or neuronal signalling in ways that are unrelated to ALS disease activity remain unanswered questions at present. Consequently, the observed reduction in NDE-derived TDP-43 may be more nuanced than a rescue of motor neuron-related changes alone. Again, this does not invalidate the finding. It simply means that the biological interpretation remains uncertain.

Pathologists frequently encounter situations where surrogate markers do not accurately reflect what is happening within the target organ. Iron metabolism provides a useful analogy. A patient may be anaemic and systemically iron deficient while simultaneously accumulating excess iron within the brain. Peripheral measurements and end-organ tissue pathology do not always move in parallel. The same principle applies here. Changes in a circulating biomarker should not automatically be assumed to represent beneficial changes in the tissues that matter most.

None of this should be viewed as criticism of the PRIME-C programme. The collection of exploratory biomarker data is exactly what early-phase studies should be doing. These findings may ultimately prove highly informative and could contribute to the development of future pharmacodynamic biomarkers. The important point is that exploratory biomarkers should be reported as data, not conclusions. A reduction in NDE-derived TDP-43 is an interesting observation. It suggests that the drug is influencing biology. What it does not yet tell us is whether that biological change is beneficial, harmful, or unrelated to disease progression. Those answers will require correlation with clinical outcomes and, ultimately, confirmation in larger studies. The Phase 2 data are encouraging and deserving of further investigation. But until the biological meaning of extracellular vesicle TDP-43 is established, caution is warranted. Biomarkers are most useful when interpreted within a validated context of use. Without that context, changes in a biomarker remain observations rather than evidence of therapeutic success.

As the field looks ahead to Phase 3, the focus should remain on learning from these exploratory measures rather than over-interpreting them. The data are valuable. The conclusions, however, must wait. Exploratory biomarkers are valuable because they help us learn. They become truly useful only when their relationship to disease biology and clinical outcomes has been established. Until then, exploratory biomarkers should be reported as data, not conclusions.

## Data Availability

No datasets were generated or analysed during the current study.

## Abbreviations

ALS: Amyotrophic lateral sclerosis
EV: Extracellular vesicle
NDE: Neuron-derived extracellular vesicle
TDP-43: TAR DNA-binding protein 43
IBD: Inflammatory bowel disease
